# Localization of nonpalpable pulmonary nodules using CT-guided needle puncture

**DOI:** 10.1186/s12957-015-0664-9

**Published:** 2015-08-15

**Authors:** Hsian-He Hsu, Chih-Hao Shen, Wen-Chuan Tsai, Kai-Hsiung Ko, Shih-Chun Lee, Hung Chang, Tsai-Wang Huang

**Affiliations:** Department of Radiology, Tri-Service General Hospital, National Defense Medical Center, Taipei, Taiwan; Department of Internal Medicine, Tri-Service General Hospital, National Defense Medical Center, Taipei, Taiwan; Department of Pathology, Tri-Service General Hospital, National Defense Medical Center, Taipei, Taiwan; Graduate Institute of Medical Science, Tri-Service General Hospital, National Defense Medical Center, Taipei, Taiwan; Division of Thoracic Surgery, Department of Surgery, Tri-Service General Hospital, National Defense Medical Center, 325, Section 2, Cheng-Kung Road, Taipei, 114 Taiwan

## Abstract

**Background:**

Surgical resection of small pulmonary nodule is challenging via thoracoscopic procedure. We describe our experience of computed tomography (CT)-guided needle puncture localization of indeterminate pulmonary nodules prior to video-assisted thoracoscopic surgery (VATS).

**Methods:**

From January 2011 to July 2014, 78 consecutive patients underwent CT-guided marking for the localization of 91 small pulmonary nodules. We retrospectively reviewed the clinical data, technical details, surgical findings and pathologic results, and complications associated with CT-guided localization.

**Results:**

Seventy-eight consecutive patients (36 men and 42 women) underwent CT-guided marking localization of 91 indeterminate pulmonary nodules (62 pure ground-glass opacity nodules, 27 part-solid nodules, and 2 solid nodules). The mean size of the nodules was 8.6 mm (3.0–23.0 mm). The mean pleural distance between the nodule and lung surface was 11.5 mm (3.0–31.3 mm). The mean procedure time of CT-guided localization was 15.2 min (8–42 min). All patients stood the procedures well without requiring conversion to open thoracotomy. Twenty-four patients (30.77 %) developed pneumothorax after the procedures. Only one patient required retention of the puncture needle introducer for air drainage. The mean visual assessment pain score was 1.7 (0–3). Fifty-seven nodules (62.63 %) were confirmed as malignances, including 45 primary lung cancer, and 34 nodules (37.37 %) were confirmed as benign lesions.

**Conclusions:**

CT-guided needle puncture can be an effective and safe procedure prior to VATS, enabling accurate resection and diagnosis of small pulmonary nodules.

## Background

As the use of computed tomography (CT) becomes widespread in clinical practice, we have increasingly encountered small or faint lesions on CT [1]. Low-dose CT greatly increases the likelihood of detection of small nodules, and 51.7 % of detected lung cancers found during baseline screening were ground-glass opacity (GGO) [2]. The accurate early diagnosis of these small nodules is challenging, even with dedicated CT, positron emission tomography–computed tomography (PET–CT), or image-guided percutaneous biopsy. Video-assisted thoracoscopic surgery (VATS) is a minimally invasive surgery for management of lung nodules, both for curative resection and diagnostic procedures. One of the major problems often encountered during VATS is localization of the target nodule, which depends on its location, size, and characteristics such as nodule consistency. Further, when small nodules are located more than 2 cm below the pleural surface, it is difficult for the surgeon to determine their exact location during operation [3]. Failure to localize pulmonary nodules often results in the conversion of VATS to open thoracotomy. Conversion rates have been reported to be as high as 59 % [4–6]. Several preoperative and intraoperative techniques have been described for nodule localization when performing VATS. These procedures include metallic hookwire localization under CT guidance [7], CT-guided micro-coil [8], a localization technique using barium [9–11], and intraoperative ultrasound [12]. However, most of these procedures have some limitations. Here, we present an alternative simple method, CT-guided needle puncture, for preoperative localization of pulmonary nodules before VATS.

## Methods

This retrospective study was approved by our institutional review board of Tri-Service General Hospital (TSGHIRB No.: 1-103-05-126), and written informed consent was not required because of strict maintenance of patient anonymity and the observational nature of the study.

### Patients and procedures

From January 2011 to July 2014, 78 consecutive patients with pulmonary nodules detected by CT scan (incidental findings, follow up because of previous malignancy, or underlying diseases) were included. Clinical parameters, including age, sex, smoking status, histology, and stage, were recorded for each patient. The characteristics of CT findings were recorded for each lesion: (a) lesion size, (b) location, (c) density, and (d) lesion distance from pleural distance (PD). All CT images were evaluated in consensus by two chest radiologists (H.H.H. and K.H.K., with 23 and 7 years of experience, respectively). Lesion size was defined as longest lesion dimension and was measured manually with electronic calipers on our picture archiving and communication system (PACS, EBM Technologies Incorporated, Taiwan). Each nodule was classified according to its density as pure nodular GGO, part solid, or solid pattern. GGO was defined as a hazy increase in lung density without obscuration of the underlying bronchial or vascular structures. A nodule was classified as part solid if it contained patches that completely obscured the lung parenchyma. We defined a solid nodule as a nodule that completely obscures the entire lung parenchyma within it.

Pulmonary nodules were followed with high-resolution CT over an interval of 3 to 6 months. Indications of tissue diagnosis for these patients included increasing nodule size, increasing soft tissue component, and underlying malignancy. Obtaining diagnostic tissue before surgery was difficult for all of these patients after consultation of radiology physicians. The nodules were not amenable for preoperative tissue diagnosis because of small lesion (less than 1 cm), location (near the vessel), and patient’s concern (refuse biopsy).

Preoperative studies included PET–CT, abdominal ultrasound, and cardiopulmonary function. All the patients gave informed consent before the procedures. The technical details, operative findings, and pathological features of nodules were evaluated.

**Fig. 1:**
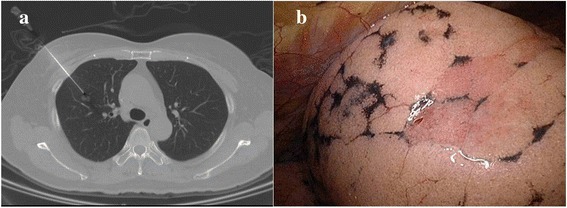
**a** The puncture needle (Temno Coaxial Introducer Needle, Care Fusion, PP1910) was inserted under the CT-guided imaging. **b** Intraoperative identification of the puncture holes at lung surface after one lung ventilation

The patient was transferred to the CT room before operation. A CT scan was performed to confirm the presence of nodules before the localization procedure. After 2 % lidocaine local injection into the puncture site of the chest wall, the introducer for a 17-gauge puncture needle (Temno Coaxial Introducer Needle PP1910, CareFusion) was inserted under CT guidance. When the introducer was inserted into the lung parenchyma (Fig. 1a) (near the lesion, but without directly puncturing the lesion to avoid tumor seeding via a puncture tract), a CT scan confirmed the location of the introducer. The needle was then inserted via the introducer. All procedures were performed by experienced radiologists (H.H.H. and K.H.K.). Afterward, the patient was transferred to the operating room. Patient positioning and preparation were the same as standard VATS. All thoracoscopic procedures were performed under general anesthesia with selective intubation through a double lumen tube to obtain ipsilateral lung collapse. The patients were placed in a lateral decubitus position. In all patients, an 11.5-mm trocar for thoracoscopy was inserted into the seventh intercostal space along the mid-axillary line. After an exploration of the pleural space, a second 11.5-mm trocar was placed according to the need for strategic visibility of the target lesion. After identification of the puncture holes (Fig. 1b), wedge resection of the target lesion was performed using an Endo-GIA™ Universal Stapler. The specimen was examined by a pathologist as a frozen section. The operations were terminated after the report of the pathological results as benign lesions. For primary lung cancer, anatomic resection and mediastinal lymph node dissection were done. We evaluated the clinicopathological data, procedure-related parameters, and complications.

### Statistical analysis

Data were entered into a spreadsheet program (Excel, Microsoft, Redmond, WA, USA). All analyses were performed using commercially available software (SPSS, version 12.2, 2004; SPSS, Chicago, IL, USA). The descriptive data were expressed as means ± standard deviation. Student’s *t* test was used to analyze continuous variables. The *χ*^2^ test was used to compare categorical variables between groups. Results were regarded as significant when *p* < 0.05.

## Results

Seventy-eight consecutive patients (36 men and 42 women; mean age 57.24 ± 9.40 years) underwent CT-guided needle puncture for the localization of 91 pulmonary nodules (Table 1). Nine patients had more than one nodule (six patients had two nodules, two patient had three nodules, one patient had four nodules). Eleven patients had an underlying malignancy, and 2 patients had sarcoidosis. The nodules included 62 pure GGO nodules, 27 part-solid nodules, and 2 solid nodules. The mean size of the nodules was 8.6 mm (range, 3.0–23.0 mm). The PD between the nodule and the lung surface was 11.5 mm (range, 3.0–31.3 mm). The mean procedure time for CT-guided localization was 15.2 min (range, 8–42 min). Twenty-five nodules had more than one puncture. The mean visual assessment score of pain for the patients after the procedures was 1.7 (0–3). All the patients tolerated the pain without any analgesics before surgery (Table 2). Twenty-four patients (30.77 %) developed pneumothorax after the procedures. There were no risk factors significantly associated with the occurrence of pneumothorax (Table 3). Only one patient (1.28 %) required retention of the puncture needle introducer for air drainage. There was no additional chest intubation. The patients were transferred to the operating room within 30 min, and the VATS were performed within 1 h after CT-guided localization. There was no conversion to thoracotomy. The lesions were identified easily by locating the puncture hole after deflation of the lung. The identification of the puncture hole was achieved immediately during the VATS. Finger palpation of the specimen was done after wedge resection of the target lung parenchyma. After identification of the nodules, the specimens were sent to the pathological department for pathological examination. All nodules were diagnosed definitively (Table 4). Fifty-seven nodules (62.63 %) were confirmed as malignancies, including 45 invasive adenocarcinoma, 7 minimally invasive adenocarcinoma, 3 atypical adenomatous hyperplasia, and 2 metastatic lesions. Thirty-four nodules (37.37 %) were confirmed as benign lesions, including 7 chronic granulomatous inflammation (three of these patients had underlying sarcoidosis), 1 harmatoma, and 1 sclerosing hemangioma. Forty-five patients who had confirmed diagnosis of invasive adenocarcinoma underwent lobectomy and lymph node dissection. The final pathological stages of these patients were stage IA.

**Table 1:** Clinical and CT characteristics of 91 nodules in 78 patients

Variables	
Male/female	36/42
Mean age (years)	57.24 ± 9.40
Size (mm)	Mean, 8.6; range, 3.0–23.0
< 10	56 (61.5)
10–20	32 (35.2)
≥ 20	3 (3.3)
Nodule from pleural distance (mm)	Mean, 11.5; range, 3.0–31.3
< 10	44 (48.3)
10–20	34 (37.4)
≥ 20	13 (14.3)
Location	
RUL	27 (29.7)
RML	5 (5.5)
RLL	16 (17.6)
LUL	25 (27.4)
LLL	18 (19.8)
Nodule density	
Pure GGO	62 (68.1)
Part-solid	27 (29.7)
Solid	2 (2.2)

Numbers in parentheses are percentages

*RUL* right upper lobe, *RML* right middle lobe, *RLL* right lower lobe, *LUL* left upper lobe, *LLL* left lower lobe, *GGO* ground-glass opacity

**Table 2:** Procedure associated complications and results

CT-guided marking complications (rate)	24 (26.4 %)
Asymptomatic pneumothorax	23 (29.49 %)
Symptomatic pneumothorax	1 (1.28 %)
Conversion to thoracotomy (rate)	0
Mean procedure time (minutes)	15.2 (8–42)
Needle puncture >1	25
VAS pain score	1.7 (0–3)

*VAS* visual assessment pain score

**Table 3:** Possible risk factors for pneumothorax

Factor	OR (95 % CI)	*p* value
Sex	1.280 (0.417–3.935)	0.666
Age (>65 years)	0.908 (0.235–3.505)	0.889
Location	0.861 (0.275–2.680)	0.476
Puncture	1.470 (0.509–4.243)	0.476
Smoking	1.375 (0.257–7.353)	0.710
PD	0.525 (0.227–1.211)	0.131

*PD* pleural distance

**Table 4:** Histopathologic results of indeterminate nodules

Variables	
Malignancy (%)	57 (62.63)
Adenocarcinoma, invasive	45
MIA	7
AAH	3
Metastasis	2
Benign (%)	34 (37.36)
Fibrosis	7
Inflammation	18
Organized pneumonia	1
Harmatoma	1
Sclerosing hemangioma	1
Chronic granulomatous inflammation	7

*MIA* minimal invasive adenocarcinoma, *AAH* atypical adenomatous hyperplasia

## Discussion

Lung cancer is the leading cause of cancer-related death worldwide. Early detection of lung cancer resulted in better prognosis. VATS is currently the preferred surgical procedure in dealing with lung tumor. Unfortunately, during thoracoscopic procedure, surgeons may have difficulty in detecting smaller and invisible nodules which were not palpable with endoscopic instruments. A conversion from VATS to thoracotomy is sometimes prescribed after failure to localize these lesions [9]. To reduce the rate of conversion to open surgery, several techniques have been developed to help the surgeon localize small nodules during thoracoscopic resection. The details of previous localization methods are summarized in Table 5. In this study, we present an alternative simple method, CT-guided needle puncture, for preoperative localization of pulmonary nodules before VATS procedures.

**Table 5:** Summary of localization procedures

Method	Advantage	Disadvantage	Ref.
CT-guided hookwire	Safe, fast	Chest wall pain, pneumothorax	[7, 13–15]
	Low complication rate	Dislodgement	
CT-guided coil	Same as above	Lung parenchyma damage	[8]
		Coil migration	
CT-guided barium spray	Same as above	Inflammatory reaction of tissue	[9–11]
Intraoperative ultrasound	Quick	Difficult for emphysematous lung	[12, 18, 19]
	More affordable	For lesion more than 1 cm	
	Less invasive	Operator dependent	
Methylene blue	Simple	Diffuse into surrounding lung	[19, 20]
	Inexpensive		
Fluoroscopic-aided contrast medium	Adequate margins of resections on fluoroscopic imaging	Contrast allergy	[21–23]
		Radiation exposure	
		Pneumothorax	
Radio-guided thoracoscopic surgery	Real-time verifying stapled margin	Gamma ray detector	[24]
		Diffusion or pleural spillage	
		Short half-life	
Bronchoscopic metallic coil marking	Avoid pneumothorax, secondary hematoma, and the intravascular injection of substances originating in needling	Ultrathin bronchoscope	[25]
		C-arm use	
		Metallic allergy	
		Coil migration, cost	
CT-guided puncture	No dye, radiotracer, or contrast medium	Cooperation with radiologist	This study
	No migration	Technique dependent	

*Ref* reference

The most widely used technique is percutaneous hookwire placement. The hookwire technique showed a variable sensitivity (58 to 97.6 %) and a relatively high failure rate because of the dislodgment of the wire [13–15]. Radioactive technetium [16, 17] to localize pulmonary nodules could achieve high success rate, leading to flexible scheduling of the operation room (with 6 h half-life of radionuclide). However, the center must have the equipment and radiation protection to offer this procedure. The ultrasonography localization method offers a quick, more affordable, less invasive way of localizing lesions and high sensitivities of 92.6 to 100 % [12, 18, 19]. However, it is highly operator dependent and is limited by the emphysematous lungs. Methylene blue staining of the nodules provides an accurate method for localizing pulmonary nodules [19, 20]. In some cases of methylene blue localization, a tendency to diffuse rapidly into the surrounding lung parenchyma after dye injection was observed [19, 20]. It obstructed the use of this procedure. Fluoroscopic-aided resection using contrast media also yielded high success rates [21–23]. CT-guided percutaneous transthoracic barium localization can be an effective and convenient preoperative localization procedure. However, several studies have reported that barium can provoke a mild acute inflammatory and edematous reaction and may make the pathological diagnosis difficult [9–11]. Radio-guided localization of pulmonary nodules is a feasible and quick procedure with a high successful rate [24]. However, spillage to the pleural space can lead to a wider area of radioactivity, which reduces the precision of the resection. In addition, the requirement for use of intraoperative gamma probes and the potential harmfulness of the radiotracer may limit the application of this technique. CT-guided bronchoscopic metallic coil marking might be a useful method for fluoroscopy-assisted thoracoscopic resection of pulmonary nodules [25]. The advantage of this procedure can avoid complications such as pneumothorax and hematoma because of transbronchial route administration of metallic coil. However, this technique may not be applicable for lesions near the hilum of the lung. It also resulted in coil migration. In addition, the cost of coil and ultra-thin bronchoscope is expensive.

In this study, we show that CT-guided needle puncture is a simple, alternative procedure for localization of pulmonary nodules before VATS. There is no requirement for involvement of additional facilities such as ultrasound, radiotracer, barium, or contrast injection. All of these nodules in our patients were detectable by CT-guided needle punctures. There was no instance of conversion to open thoracotomy. Initially, we used a 20-gauge needle, but in some patients, the puncture holes could only be found after careful inspection. In some patients, we indentified the puncture hole after inflation/deflation of the lung (air bubble emerged from the puncture hole). With accumulation of the experiences, the puncture hole could be visualized easily with use of 17-gauge needle. The occurrence of pneumothorax was not significantly different between different needle sizes. However, because of the small size of this study, verification of this relationship may require additional patient data. In our preliminary experiences, there was no life-threatening pneumothorax. It was no necessary to put chest tube even if development with symptomatic pneumothorax even if there is development of symptomatic pneumothorax. In addition, a small percentage of pneumothorax may provide the clue of precise puncture of lung parenchyma.

**Fig. 2:**
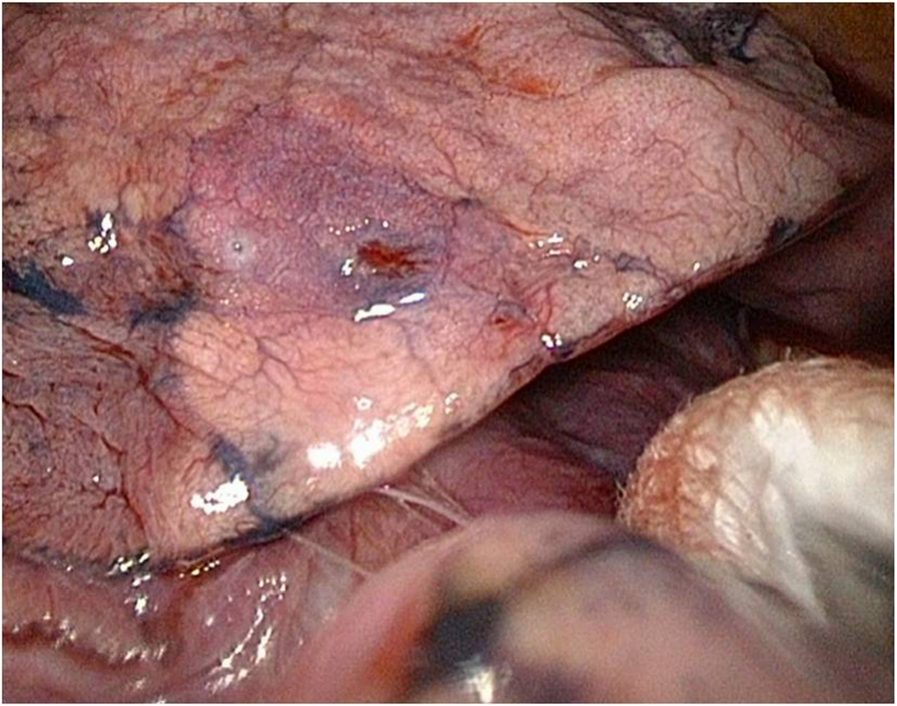
This pulmonary hemorrhage was useful in identification of the target lesion

The location of nodule did not affect this procedure. The PD of the nodule did not affect the occurrence of pneumothorax. For deeper lesion, we often made more than one puncture for one lesion in the beginning of this study. However, there was no significant difference in the puncture number (one puncture hole group PD, 1.14 ± 0.69 mm; two puncture holes group PD, 1.15 ± 0.81, *p* = 0.99). The location of the nodules (central or peripheral lesion) had no significant influence on the attempt of puncture number (Table 6). For deeper nodules, it is possible to obtain two-dimensional localization when performing wedge resection of the lung parenchyma by using two punctures from different directions. This concept provides precise localization of nodules for VATS. In addition, pulmonary hemorrhage could result in pigmentation of the lung surface; this was useful in identification of the target lesion (Fig. 2). At initial practices, the patients were transferred to the operating room after localization procedures within half an hour. After that, we found that the puncture holes could still be identified even if the surgery was started 4 h later. With accumulation of more experiences, we propose that this procedure provides available time frame between labeling of nodule and surgery.

**Table 6:** Comparison of one versus two punctures

Variables	Single puncture group *(n* = 66)	Two punctures group *(n* = 25)	*p* value*
Age (year)	56.27 ± 8.98	59.80 ± 10.19	0.11
PD (cm)	1.14 ± 0.69	1.15 ± 0.81	0.99
Location			
Central	23	5	0.67
Peripheral	43	20	

* p< 0.05

The advantage of this procedure is that the pre-localization CT scan can confirm the presence of nodules. Two patients avoided a VATS procedure after the pre-localization CT scan showed decreased size or disappearance of the lesion. This procedure is performed by radiologists and is very similar to the procedure for a CT-guided core needle biopsy, which most radiologists are very familiar, so that there was no additional learning curve or difficulty in performing this procedure. In addition, the procedure does not require additional facilities as do procedures such as radiotracer, contrast injection, coil insertion, or special ultrasound. There was no additional radiation exposure for the patients or physician during the operation and no necessity of a gamma probe to detection.

The limitations of this study are its small size and that it was a single-institution retrospective study. More convinced data should be obtained via including more patients and long-term follow-up. In addition, the cooperation of the radiologist and the use of available operating rooms are important. Prospective clinical trials of patients with indeterminate pulmonary nodules should be conducted to clarify the need for two punctures and the feasibility of using the technique for deeper lesions.

## Conclusions

In conclusion, CT-guided needle puncture localization is feasible and simple for indeterminate pulmonary nodules before VATS. The procedure can result in a high success, low complications, and low cost without any additional facilities.
